# Intrasession and Between-Visit Variability of Retinal Vessel Density Values Measured with OCT Angiography in Diabetic Patients

**DOI:** 10.1038/s41598-018-28994-7

**Published:** 2018-07-13

**Authors:** Cecília Czakó, Gábor Sándor, Mónika Ecsedy, Zsuzsa Récsán, Hajnalka Horváth, Zsuzsanna Szepessy, Zoltán Zsolt Nagy, Illés Kovács

**Affiliations:** Department of Ophthalmology, Semmelweis University, 26 Üllői Street, 1085 Budapest, Hungary

## Abstract

In clinical practice the measurement error of an instrument has special importance in analyzing and interpreting data, and acknowledging limitations. The purpose of this study was to evaluate intrasession and between-visit reproducibility of OCT angiography measurements in diabetic patients. A total of 54 eyes of 27 diabetic patients underwent OCT angiography imaging. Foveal avascular zone (FAZ) area and superficial retinal vessel density (VD) at 3 mm were calculated using the AngioAnalytics software. Three consecutive images were acquired at first visit and one image 1 month later. Intrasession and between-visit reproducibility of parameters were characterized by intraclass correlation coefficient (ICC), coefficient of variation (CV), and coefficient of repeatability (CR) values. We measured excellent (>0.90) ICC values both in intrasession and between-visit comparisons. CV was higher for the FAZ area compared to VD both in intrasession (7.79% vs. 2.87%) and in between-visit (12.33% vs. 2.95%) comparisons. Between-visit CR value for VD was 4.53% (95% CI: 3.72–5.79%). These data suggest that OCT angiography shows excellent repeatability in diabetic patients, indicating that this non-invasive technology might be suitable for longitudinal assessment of microvascular complications.

## Introduction

Diabetic retinopathy (DR) is a common microvascular complication that develops in almost 80% of diabetic patients after 15 years^1,2^. According to a recent study each year in the duration of diabetes represents a 6% increase in the onset of diabetic retinopathy^3^ and DR has a significant effect on life quality, particularly at more advanced stages with vision-threatening complications (ischemic maculopathy and clinically significant macular edema)^4^. Microvascular damage in diabetes leads to capillary nonperfusion and ischemia, upregulating the production of vascular endothelial growth factor (VEGF) subsequently leading to pathologic neovascularization and increased vascular permeability^5^. Diabetic macular edema (DME), which can develop at any stage of DR is responsible for 4.8% of blindness worldwide^6^.

Better identification of diabetic patients at high risk of vision-threatening complications is crucial for the prevention of vision loss. Ocular imaging by fluorescein angiography and optical coherence tomography has been playing a significant role in the visualization of retinal vascular perfusion and macular edema, respectively. Optical coherence tomography angiography (OCTA) is a non-invasive imaging technique that is able to visualize separately the different retinal and choroidal vascular layers using motion contrast technology to detect blood flow without intravenous dye injection^7^. Since the availability of OCTA, numerous studies have reported the changes of retinal microvasculature of patients with diabetes mellitus such as vascular remodeling, enlarged FAZ, and capillary tortuosity and dilation^8–17^.

In clinical practice the measurement error of an instrument has special importance that depends on the context in which the measurements in question are to be used. Usually, the same degree of measurement error that is acceptable in a comparative study, such as a clinical trial, may be too large in individual patient management, such as in a follow-up or the assessment of a therapeutic response. Moreover, it is important to know the measurement error of an instrument in designing clinical trials, analyzing and interpreting data, and acknowledging limitations – all of which are essential for clinical research. Although the high accuracy and reproducibility of OCTA parameters have already been described in normal subjects^18–25^, there is a lack of data on the reproducibility of OCTA measurements in diabetic patients. The purpose of this study was to evaluate the intrasession and between-visit reproducibility of quantitative retinal microvasculature parameters using OCTA in patients with non-proliferative diabetic retinopathy.

## Methods

**Figure 1:**
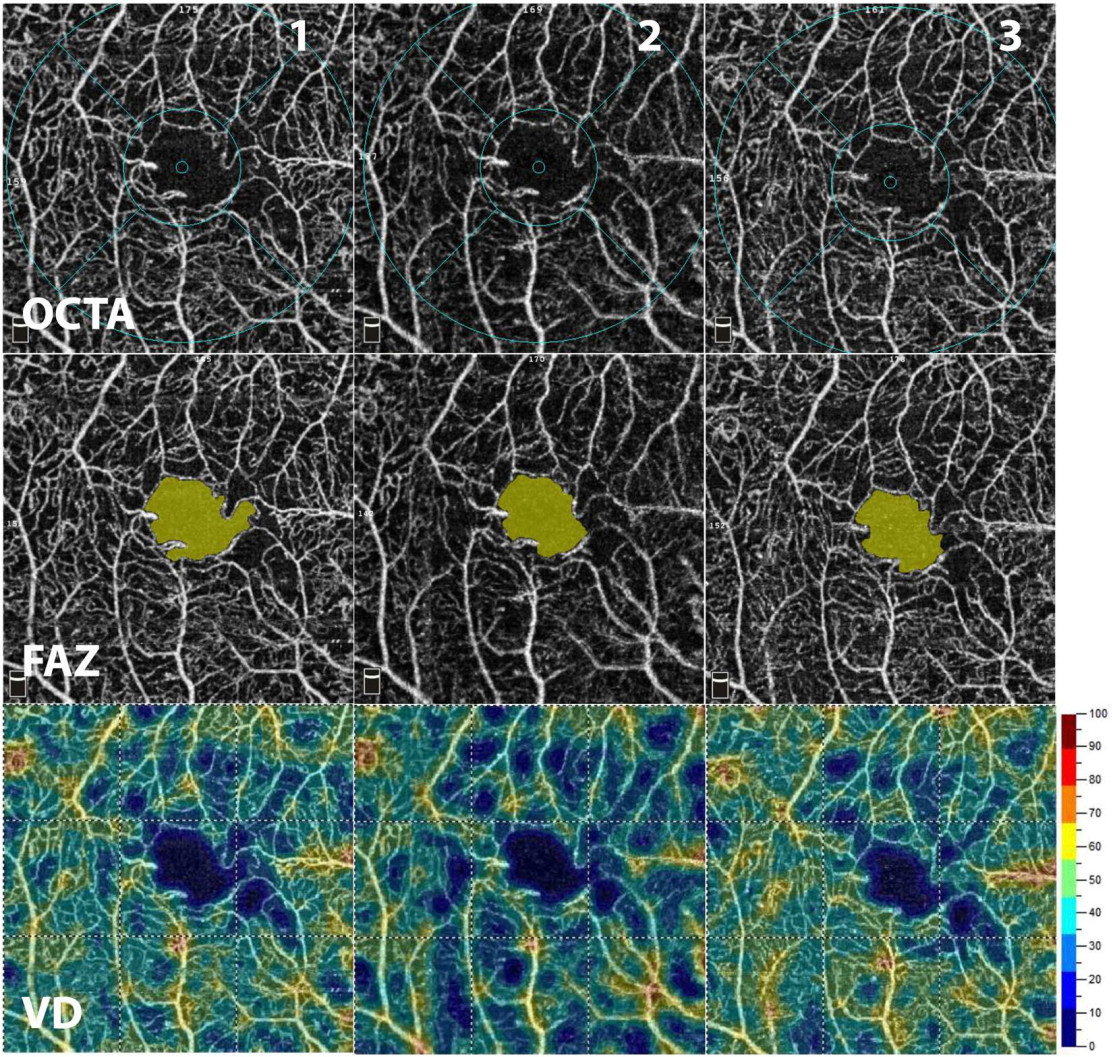
*En face* OCTA imaging of the superficial retinal capillary plexus (SCP) using the automated AngioAnalytics software in a diabetic subject after the acquisition of three consecutive images from the same subject (1, 2, 3 – top row). Foveal avascular zone (FAZ – middle row) area and vessel density (VD – bottom row) of the SCP were measured using the non-flow detection and flow density tool show substantial fluctuation across the images.

In this prospective, observational cross-sectional institutional study, a total of 54 eyes of 27 subjects (15 male and 12 female, mean age: 59.57 ± 13.22 years) with type 1 and type 2 diabetes were recruited from the outpatient clinic of the Department of Ophthalmology at Semmelweis University. The study followed the tenets of the Declaration of Helsinki, applicable national and local requirements, and was approved prospectively by the Ethical Review Board for Human Research of the National Drug Agency. All patients signed their written informed consent. Diabetic patients without any retinopathy and patients with clinically detectable retinopathy, as defined by the International Clinical Diabetic Retinopathy Disease Severity Scale of the American Academy of Ophthalmology, were recruited in the study. Exclusion criteria were the following: any history of intraocular surgery, other ocular disease (such as age-related macular degeneration, glaucoma, vitreomacular disease), previous intraocular anti-VEGF, steroid or laser treatment, the presence of clinically significant lens opacities, or refractive error >6 diopters. All subjects underwent a comprehensive ophthalmic examination including slit lamp and fundus examinations using indirect ophthalmoscope. Optical coherence tomographic angiography imaging was performed using the AngioVue OCTA system (RTVue-XR Avanti, OptoVue, Fremont, CA, USA) with an SSADA (split-spectrum amplitude decorrelation angiography) software algorithm. This device obtains volumetric scans of 304 × 304 A-scans at 70 000 A-scans per second in approximately 3.0 seconds. Full retinal thickness was measured in the central 1.0 mm area. Superficial vessel density (VD) was evaluated in the central 3 mm and parafoveal area, and foveal avascular zone (FAZ) area was measured using the built-in AngioAnalytics software of OptoVue system with an automated segmentation algorithm (Fig. 1). The FAZ area corresponded to the non-flow area and was measured in square millimeters (mm^2^) using the automatic non-flow tool of the software. In this study we analyzed retinal vessel density in the central 3 mm diameter area as the current version of the AngioAnalytics software acquires scans with the highest resolution in the central 3 × 3 mm area enabling confident evaluation of fine vessels in this region of the macula. In addition, we evaluated changes solely in the superficial capillary plexus, with the level at which the segmentation line was positioned with an inner boundary being 3 µm below the internal limiting membrane (ILM) and an outer boundary 15 µm below the inner plexiform layer (IPL), as quantitative analysis of the deep capillary plexus is unreliable using the current AngioVue software due to projection artefacts. Moreover, images with pronounced diabetic macular edema, which generated consecutive segmentation errors at the level of the superficial vascular plexus, were excluded from the study. As a result, no segmentation line needed manual modification and positional change of the retinal blood vessels due to DME was not observed. Since image artefacts – such as movement artefacts (vessel doubling, white line artefacts, noise or vessel discontinuities), projection artefacts and segmentation errors – can provide false and misleading information, in patients with initially poor images, we repeated the scans until good image quality could be obtained. The selection of images for the purpose of exclusion was conducted by one individual trained examiner, with poor quality images, which were excluded from the analysis being defined as those: with a Signal Strength Index (SSI) below 50; autosegmentation alignment errors on OCT B-scans at the level of the superficial capillary plexus induced by DME; and, images with a presence of artefacts, such as double vessel pattern and dark areas from blinks or media opacities that obscure vessel signal. Although the recommended cut-off value of the SSI is greater than 39 on a scale from 0 to 100 according to the manufacturer’s recommendation, in previous research papers the generally accepted threshold value was 50; thus in our study we have adopted this limit. Each study subject underwent two sessions of imaging; in the first session three OCTA images were obtained and one month later, in the second session one OCTA image was acquired.

### Data availability

all data analyzed during this study are included in this published article.

### Statistical analysis

Statistical analysis was performed with SPSS software (version 23.0, IBM, Armonk, NY, USA). The Shapiro-Wilks W test was used to test the normality of the data.

Sample size was determined *a priori* by statistical power calculation (power 0.90; p = 0.05) using data from previous studies at our institution and the method proposed by Lu *et al*. for repeated measurements^26^. The minimum number of eyes to enroll in this study was calculated to be 37 eyes with a maximum allowed difference of 5% in vessel density values between two consecutive measurements on the same subject.

Intrasession and between-visit reproducibility were characterized by the corresponding intraclass correlation coefficient (ICC) and within-subject coefficient of variation (CV) values. Intrasession and between-visit intraclass correlation coefficients (ICC) were calculated using two-way mixed-effects models with their 95% confidence intervals on absolute agreement^27^. The ICCs could range from 0 to 1, with a higher value indicating better reliability and an ICC above 0.9 indicates excellent clinical repeatability^28^. Intrasession and between-visit CVs with their corresponding 95% confidence intervals were calculated according to the procedure described by Bland and Altman^29^. For this purpose, the within-subject standard deviation (Sw) was calculated as the square root of the within-subject mean square of error in a two-way mixed-effects model, and then CV was determined as 100× within-subject standard deviation/overall mean^30^. Finally, Bland- Altman plots were created by plotting the coefficient of repeatability (CR), which is a useful index that quantifies measurement error in the same units as that used in the measurement tool. The CR of an instrument corresponds to the 95% limits of agreement on Bland-Altman plots and contains 95% of differences between repeated measurements on same subjects. The CR is the value below which the absolute differences between two measurements would lie with 0.95 probability^31–33^ and can be calculated by multiplying the within-subject standard deviation (Sw) by 2.77 (√ 2 times 1.96)^32,33^.

In all statistical analyses, a P-value of less than 0.05 was considered to be statistically significant.

## Results

Patients characteristics are summarized in Table 1. Considering OCTA measurements, there was no significant difference in the mean FAZ area between the two visits (0.30 ± 0.11 mm^2^ vs. 0.31 ± 0.11 mm^2^; p > 0.05). Similarly, there was no significant difference in 3.0 mm vessel density (47.94 ± 3.85% vs. 47.70 ± 3.96%; p > 0.05) and in parafoveal vessel density (49.48 ± 4.08 vs. 49.26 ± 4.19%; p > 0.05) between the two visits. However, as an indicator of intrasession variability of measurements, fluctuation in OCTA metrics could have been observed on images taken consecutively from the same subject as one can see on the representative Fig. 1.

**Table 1:** Clinical characteristics of the study population.

Ocular characteristics (n = 54)	
Axial length (mm)	23.01 ± 0.65
Visual acuity	0.83 ± 0.27
Diabetic retinopathy	
No	20
Mild	21
Moderate	11
Severe	2
Diabetic macular edema (Y/N)	19/35

Note: Data shown as mean ± SD.

Intraclass correlation coefficient values were excellent (>0.90) both in intrasession and between-visit comparisons of measurements (Table 2). However, CV for the FAZ area was significantly higher than CV values of vessel density in the 3.0 mm macular area and in the parafoveal ring, both in intrasession (p < 0.01; Table 2) as well as in between-visit comparisons (p < 0.01; Table 2). By comparing intrasession and the corresponding between-visit CV values of the evaluated parameters, we found no significant differences between the results (p > 0.05 for all parameters).

**Table 2:** Intrasession and between-visit repeatability of the different OCTA metrics expressed by intraclass correlation coefficient (ICC) and coefficient of variation (CV) values.

Intrasession repeatability	ICC	95% CI	CV	95% CI
FAZ area	0.97	0.95–0.98	7.79*	4.66–10.92
VD at 3 mm area	0.97	0.95–0.98	2.87	1.79–3.95
VD at parafoveal ring	0.96	0.93–0.98	3.55	3.02–4.08
Between-visit repeatability				
FAZ area	0.94	0.89–0.97	12.33*	9.34–15.32
VD at 3 mm area	0.91	0.83–0.95	2.95	1.69–3.21
VD at parafoveal ring	0.91	0.83–0.95	4.03	3.02–5.04

Note: FAZ: foveal avascular zone; VD: vessel density; CI: Confidence Interval, *p < 0.01 between FAZ area and VD.

**Figure 2:**
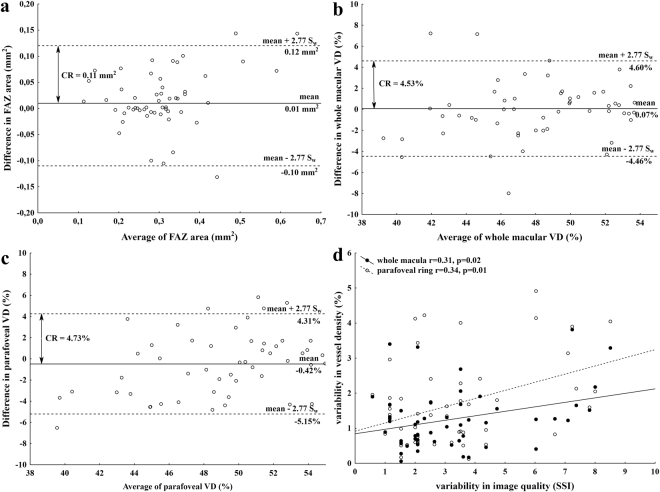
Bland-Altman plots showing between-visit reproducibility of foveal avascular zone (FAZ) area (**a**) vessel density (VD) at 3 mm (**b**) and parafoveal VD (**c**) with the 95% limits of agreement (dashed line) that correspond to the coefficient of repeatability (CR) values. Intrasession variability of image quality significantly correlated with the variability in vessel density values (**d**).

Next, Bland-Altman analysis was used to describe between-visit agreement of the repeated measurements. As it is shown on the Bland-Altman plots, the mean difference was 0.07% for the VD in the whole macula and 0.42% for VD in the parafoveal ring and 0.01 mm^2^ for the FAZ area. In addition, the presence of any proportional error during the repeated measurements was assessed by drawing a regression line on the differences seen in Fig. 2b and c. These regression analyses did not show any proportionality effect between mean values of VD and the difference in VD either in the whole macular area (p = 0.13, p = 0.36) or in the parafoveal ring (r = 0.27, p = 0.08; regression lines not shown on Fig. 2b and c due to lack of significance).

Given the possible effect of image quality on the repeatability of measurements, we additionally assessed the variability of image quality expressed by their corresponding SSI values and its influence on vessel density measurements. The intrasession CV value for SSI was 3.59% (95% CI: 2.91–4.27%) suggesting that image quality had excellent reproducibility in this study. However, by analyzing the triplicate measurements from the first visit, we found a significant correlation between the variability of SSI and the variability of vessel density values in the whole macula (r = 0.31, 95% CI: 0.05–0.58; p = 0.02), and also in the parafoveal ring (r = 0.34, 95% CI: 0.08–0.61; p = 0.01; Fig. 2d).

Finally, in order to evaluate the possible effect of macular edema on image quality and OCTA metrics, we compared image quality in eyes with (n = 21) and without (n = 33) macular edema. We found that image quality was significantly lower in eyes with macular edema compared to those without (Table 3), although neither intrasession nor between-visit variability of SSI values were affected by the presence of diabetic macular edema (Table 3). In eyes with macular edema, repeatability of foveal avascular zone measurements was significantly worse than in eyes without diabetic edema, while the presence of DME did not show any effect on the repeatability of vessel density measurements (Table 4).

**Table 3:** Image quality expressed by Signal Strength Index (SSI) in eyes without CMO and with CMO at the first and the second visit.

	Eyes without CMO (n = 37)	Eyes with CMO (n = 17)	P
First visit			
SSI 1	68.17 ± 7.70	62.06 ± 7.31	0.008
SSI 2	68.17 ± 8.03	61.53 ± 6.74	0.004
SSI 3	66.89 ± 8.04	59.65 ± 8.44	0.004
Intrasession variability of SSI	3.20 ± 2.10	3.46 ± 2.02	0.67
Second Visit			
SSI	69.05 ± 8.75	60.41 ± 12.23	0.004
Between-visit variability of SSI	3.51 ± 3.11	3.93 ± 2.62	0.64

Note: Data expressed as mean ± SD, P: Student’s t-test on independent sample.

**Table 4:** Intrasession and between-visit coefficient of variability values in eyes with and without CMO.

	Eyes without CMO (n = 37)	Eyes with CMO (n = 17)	P
Intrasession coefficient of variability			
Foveal avascular zone	5.83 ± 5.02	11.78 ± 13.25	0.02
3 mm vessel density	2.82 ± 1.71	2.33 ± 2.05	0.36
Parafoveal vessel density	3.49 ± 2.68	3.75 ± 3.33	0.76
Between-visit coefficient of variability			
Foveal avascular zone	8.88 ± 9.49	20.3 ± 26.4	0.02
3 mm vessel density	2.51 ± 2.50	3.92 ± 3.75	0.11
Parafoveal vessel density	3.62 ± 3.89	5.03 ± 3.13	0.20

Note: Data expressed as mean ± SD, P: Student’s t-test on independent samples.

## Discussion

In the current study we investigated the intrasession and between-visit reproducibility of AngioFlow measures determined by the AngioVue OCTA system in diabetic patients. We found that both intrasession and between-visit variability of data are substantially larger when analyzing the FAZ area compared to variability of vessel density values in the macula. Moreover, in eyes with macular edema, repeatability of foveal avascular zone measurements was significantly worse than in eyes without diabetic edema, while the presence of DME did not show any effect on the repeatability of vessel density measurements. This study quantified the reproducibility of OCTA metrics in diabetic patients showing better reproducibility of vessel density values compared to FAZ area, suggesting that it is the preferable variable to assess microvascular alterations in this population.

Similarly to previous studies on healthy subjects^18,34^, we have found that OCT angiography provides excellent intrasession and between-visit reproducibility of measurements in the same subject. In this study we demonstrated in diabetic patients that both intrasession and between-visit repeatability of OCTA measurements are comparable, however, both intrasession and between-visit CV values were higher for the foveal avascular zone area compared to CV values of the macular vessel density.

In addition, we have provided coefficient of repeatability (CR) values for different OCTA metrics as well, since these data may have direct implications on the follow-up of this population. The coefficient of repeatability can be interpreted as the smallest change in OCTA metrics that represents true change^32,34^. This parameter accounts for both random and systematic error in its scores and there is a consensus on the advantage of considering measurement error as calculated by the CR over the Intraclass Correlation Coefficient (ICC)^32,33^. In our study, the between-visit CR of the vessel density in the 3 mm macular area was similar (±4.53%) to that for the parafoveal ring (±4.73%). The CR (±4.53%) means, that vessel density values in any individual patients would be of this scale, being within 4.53% of this bias. Based on the CR of the 3 mm macular area, a clinician using the retinal vessel density in a diabetic patient would need to measure a change of at least 4.53% at re-assessment to be 95% confident that the patient had, in fact, progression in diabetic microangiopathy. A change of less than 4.53% might simply be due to the measurement inaccuracy of OCTA, which is unable to reliably detect change of less than 4.53%. Our results are in a good harmony with the findings of a previous study on healthy subjects, where a CR of 4.1–4.64% for the whole scan and a CR of 4.4–4.85% for the parafoveal VD in the 3 × 3-mm OCTA images were calculated^23,34^.

Diabetic retinopathy was previously mainly associated with visible pathology of the retinal vasculature; however, OCT angiography is a sensitive tool that can indicate subtle microvascular damage even before clinical vascular abnormalities can be seen. OCTA technology enables us to study, both qualitatively and quantitatively, the retinal microcirculation^35^ with high reproducibility and reliability^36^. The AngioAnalytics flow density map software released by the AngioVue system allows the quantification of flow area, FAZ area, and flow density around the fovea. Vessel density measurements estimate the degree of capillary loss over an area as a percentage of the total evaluated area^35^, however, OCTA cannot directly detect leakage from the retinal blood vessels and cannot image early signs of peripheral retinal nonperfusion^37^. In this study we analyzed retinal vessel density in the macular area focusing on the changes in superficial retinal vascular bed as quantitative analysis of the deep capillary plexus is not reliable using the AngioVue software due to projection artefacts^38^. Apart from confirming previous results on the negative effect of projection artefacts on quantitative results^39^, we also confirmed findings that the variability of vessel density measurements is significantly related to the variability of SSI values^23^, showing the importance of high quality image acquisition during OCTA. We also found, that image quality was significantly lower in eyes with macular edema compared to those without, although neither intrasession nor between-visit variability of image quality was affected by the presence of diabetic macular edema. Cystoid macular edema in diabetes is located in different retinal layers, especially in the outer plexiform layer^40^. While the retinal layers are easily distinguishable in healthy retinas, the presence of macular edema decreases the contrast between different layers and the segmentation remains very challenging for automated methods. Although recent studies have introduced automated segmentation methods for eyes with severe macular edema, their availability remains limited^41^, thus, before quantitative analysis of OCTA images any segmentation error due to DME should be identified. The decreased reliability of AngioFlow measures due to compromised image quality and projection artefacts can be particularly important during the analysis of deep capillary plexus, which has already been described as helping in the identification of eyes at increased risk of developing DR^42^. The possible effects of segmentation error on our results could be dismissed from consideration since in this study we evaluated changes in the superficial capillary plexus, and images with misidentification of segmentation lines on OCT B-scans induced by macular edema were excluded.

Knowledge of the measurement error of an instrument is especially important both in the detection of retinal abnormalities at their earliest stages and in patient follow-up^43,44^. In addition, in designing clinical trials with OCTA, knowledge of the repeatability of measurements is crucial in order to calculate the sample size required to demonstrate disease progression or the impact of an intervention that is intended to reach a clinically relevant threshold. Repeatability of measurements refers to the variation in repeated measurements made on the same subject under identical conditions and is particularly important in clinical settings. To assess repeatability, measurements should be taken by the same instrument over a short period of time, over which the underlying value can be considered to be constant. Therefore, variability in measurements made on the same subject in a repeatability study can then be attributed only to errors due to the measurement process itself. In this study, repeated OCTA measurements made during the first visit corresponded to intrasession repeatability, while the second visit - scheduled approximately one month after the first visit - was used to assess between-visit repeatability and mirrored the usual follow-up intervals of diabetic patients (i.e. frequency of intravitreal anti-VEGF injections). Therefore, it is our opinion that the results on the repeatability of OCTA metrics can be generalized to a clinical population of diabetic patients, and thus helping the clinical care of this population.

As a limitation of the present study, the data is obtained from a cohort from a single center, which may limit the generalizability of our results to other populations. Nevertheless, we assume that given the number of eyes included in this study the conclusions drawn from our results are reliable. Another significant limitation of the study is the absence of information on the patient’s nutritional status, concomitant cardiovascular and other non-cardiovascular conditions that may contribute to the between-visit variability of OCTA parameters. Although this information would have helped elucidate the relationship between systemic factors and the repeatability of measurements, the primary purpose of this study was to assess the reliability of OCTA in detecting retinal microvascular damage to make both screening and assessment of progression more sensitive.

The fact, that the repeatability of retinal vessel density measurements was not affected by the presence of diabetic macular edema supports the assumption that the progression of microvascular damage can be evaluated accurately in different stages of non-proliferative diabetic retinopathy. However, further studies are recommended to examine whether the between-visit repeatability of OCTA measurements could be improved by using average values of consecutive measurements during each visit.

In conclusion, repeated OCT angiography measurements of macular vessel density revealed excellent repeatability, indicating that this non-invasive technology is sufficient for longitudinal assessment of microvascular complications in diabetes. However, a change of less than 4.5% in superficial vessel density might simply be due to the measurement inaccuracy of OCTA, thus a change of at least 4.5% at re-assessment is required to indicate with 95% confidence that the patient had, in fact, a true change in retinal capillary blood flow.
